# Human cord blood-derived primitive CD34-negative hematopoietic stem cells (HSCs) are myeloid-biased long-term repopulating HSCs

**DOI:** 10.1038/bcj.2015.22

**Published:** 2015-03-13

**Authors:** Y Matsuoka, K Sumide, H Kawamura, R Nakatsuka, T Fujioka, Y Sasaki, Y Sonoda

**Affiliations:** 1 Department of Stem Cell Biology and Regenerative Medicine, Graduate School of Medical Science, Kansai Medical University, Hirakata, Osaka, Japan; 2 Department of Orthopedic Surgery, Kansai Medical University, Hirakata, Osaka, Japan

Hematopoietic stem cells (HSCs) possess both self-renewal and multi-lineage differentiation abilities and maintain lifelong hematopoiesis. Recent studies have revealed that the murine HSC compartment consists of heterogeneous HSC subpopulations in terms of their lineage-biased differentiation potentials.^1, 2, 3, 4, 5, 6^ Dykstra *et al.* have recently categorized murine HSCs as α-, β-, γ- and δ-cells, according to the contribution ratio of myeloid or lymphoid cells in the repopulation assays.^1, 2^ In addition, other groups have reported that lineage-biased HSCs could be prospectively isolated by their surface immunophenotypes. For example, CD41^+^ murine HSCs have shown to possess a long-term (LT) repopulating capacity and showed a marked myeloid-biased reconstituting capacity.^3^ In addition, the murine platelet-primed von Willebrand factor (vWF)-positive HSCs have LT-myeloid-biased lineage repopulation potentials and can self-renew.^4^ Furthermore, Morita *et al.*^5^ reported that the murine HSC compartment could be segregated according to the expression levels of CD150 antigen, and CD150^high^ HSCs had the most potent self-renewal activities, as well as the the highest myeloid-biased lineage differentiation potentials. All of these reports demonstrated that myeloid-biased murine HSCs have a LT-repopulating capacity and can produce lymphoid-biased HSCs. Therefore, myeloid-biased HSCs were thought to be most primitive HSCs in the murine HSC hierarchy.^1, 2, 3, 4, 5, 6^

Conversely, it has not yet been elucidated whether the human HSC compartment consists of homogeneous or heterogeneous HSC subsets in terms of the lineage-biased differentiation potentials. It has long been believed that human HSCs and hematopoietic progenitor cells (HPCs) exist only in the CD34-positive (CD34^+^) fraction. However, we recently identified CD34-negative (CD34^−^) SCID-repopulating cells (SRCs) in the human cord blood (CB) using an intra-bone marrow injection (IBMI) technique.^7, 8, 9, 10, 11, 12, 13^ In addition, we developed high-resolution purification methods for these CB-derived CD34^−^ SRCs using 18Lineage (18Lin)-specific antibodies.^10, 11, 12, 13^ These highly purified lineage-depleted CB-derived CD34^−^ cells possessed not only SRC abilities but also colony forming cell (CFC) abilities in the methylcellulose semi-solid culture.^10, 11, 12, 13^ These observations clearly demonstrated that human CB-derived HSCs/HPCs (HSPCs) exist not only in the CD34^+^ but also in the CD34^−^ fraction. However, the functional differences between these human CD34^+/−^ HSPCs are not fully elucidated. Therefore, in this study, we precisely analyzed the differences in the differentiation potentials between CD34^+/−^ HSPCs *in vivo* and *in vitro*.

In order to compare the *in vivo* differentiation potentials of CD34^+/−^ SRCs, we first performed an SRC assay. The CB-derived 18Lin-negative (18Lin^−^) CD34^+/−^ cells, both of which contain highly purified CD34^+/−^ SRCs,^10^ were transplanted into NOD/Shi-scid/IL-2 Rγ_c_^null^ (NOG) mice using the IBMI technique. Then, the percentages of CD19^+^, CD33^+^ and other types of cells (defined as human CD45^+^, CD19^−^, CD33^−^ cells) in the human CD45^+^ cells produced from CD34^+/−^ SRCs in the mouse BMs were serially analyzed (schematically presented in Supplementary Figure S1). The human CD45^+^ cell repopulation capacities of both CD34^+/−^ SRCs were not significantly different (Figure 1a and Supplementary Figure S2). These data were consistent with our previously reported data that both CD34^+/−^ SRCs possessed comparable human CD45^+^ cell repopulation capacities.^7, 8, 9, 10, 11, 12, 13^ However, the differentiation potentials of these CD34^+/−^ SRCs with regard to the CD33^+^ myeloid cells were clearly different. These CD34^−^ SRCs showed significantly higher rates of CD33^+^ myeloid cell repopulation (Figures 1e and 2). At 5–6, 12 and 18–24 weeks after transplantation, CD34^−^ SRCs showed significantly higher percentages of CD33^+^ cells (74.4, 30.4 and 29.8%, respectively; *P*<0.01) compared with CD34^+^ SRCs (22.8, 13.1 and 17.7%, respectively) in the mouse BMs (Figures 2g–i). Surprisingly, a number of the mice that received CD34^−^ SRCs showed exclusively human CD33^+^ myeloid cell repopulation at 5 weeks after transplantation (Figure 2d and Supplementary Figure S3B). However, these CD34^−^ SRCs were not myeloid-committed progenitors. Because all of the mice received CD34^−^ SRCs showed multi-lineage human hematopoietic cell reconstitution at 18–24 weeks after transplantation (Figures 1 and 2, Supplementary Figures S2 and S3). We have previously reported that these CD34^−^ SRCs possessed secondary and tertiary (>1 year) multi-lineage reconstituting abilities as did CD34^+^ SRCs.^12^ The percentages of CD33^+^ cells in the mice that received CD34^−^ SRCs were gradually decreased from the early-to-late weeks after transplantations (Figures 2g–i), and concomitantly the percentages of CD19^+^ B-lymphoid cells increased (Figures 2j–l).

In contrast, CD34^+^ SRCs produced significantly higher percentages of CD19^+^ cells compared with CD34^−^ SRCs until 12 weeks after transplantation (Figures 2a, b, j and k). At 5–6, 12 and 18–24 weeks after transplantation, the mean percentages of CD19^+^ cells in the mouse receiving both CD34^+/−^ SRCs were 66.7 and 21.0% (*P*<0.01), 80.8 and 64.7% (*P*<0.05), and 59.7 and 65.6% (*P*=0.496), respectively (Figures 2j–l). Therefore, CD34^+^ SRCs predominantly produced CD19^+^ cells in the mouse BM at each time point (Figure 2 and Supplementary Figure S3A). These results were consistent with recently reported data.^14^

We next further analyzed the multi-lineage differentiation potentials of CD34^+/−^ SRCs. At 18–24 weeks after transplantation, mice were killed and the human hematopoietic multi-lineage reconstitutions in the mouse left tibia (injection site) were analyzed by FACS. Both CD34^+/−^ SRCs could produce comparable levels of CD34^+^ progenitor cells, CD19^+^ B lymphocytes, CD14^+^ monocytes, CD41^+^ megakaryocytes and CD3^+^ T lymphocytes in the murine BM (Figures 1b–d, f and g and Supplementary Figure S2), as we reported previously.^10, 11, 12, 13^ However, CD34^−^ SRCs produced higher percentages of CD33^+^ cells compared with those of CD34^+^ SRCs (Figures 1e and 2g–i), as above-mentioned. On the contrary, CD34^+^ SRCs produced a significantly higher percentage of CD235a^+^ cells compared with CD34^−^ SRCs (Figure 1h). Collectively, these results demonstrated, for the first time, that human CB-derived CD34^−^ SRCs are myeloid-biased SRCs.

We further analyzed *in vitro* the differentiation potentials of CD34^+/−^ HSPCs by a CFC assay and coculture with human bone marrow-derived mesenchymal stromal cell (MSC) feeders, which support human HSCs, as we recently reported.^13^ As shown in Supplementary Figure S4A, in the presence of 30% fetal calf serum (FCS) supplemented with a cocktail of cytokines, including stem cell factor, interleukin (IL)-3, granulocyte colony-stimulating factor, granulocyte/macrophage colony-stimulating factor and erythropoietin (EPO), the plating efficiency (PE) of 18Lin^−^CD34^+^ cells (69.8%) was significantly higher than that of 18Lin^-^CD34^-^ HSPCs (50.9%) (*P*<0.05). The 18Lin^-^CD34^+^ cells formed all types of colonies, including CFU-GM (61.5%), BFU-E (30.2%) and CFU-Mix (7.0%). Conversely, 18Lin^−^CD34^−^ cells formed mainly BFU-E (57.4%) and CFU-Mix (40.9%) colonies, and only a few myeloid colonies (1.7%), which is consistent with our recent data.^10, 11, 12, 13^ In addition, in order to further assess the erythroid and megakaryocyte differentiation potentials of CD34^+/−^ HSPCs, a CFC assay was performed in the presence of 10% platelet-poor plasma supplemented with TPO, EPO and IL-3. Under these conditions, the PE of 18Lin^−^CD34^−^ cells (66.9%) was significantly higher than that of 18Lin^−^CD34^+^ cells (29.4%) (*P*<0.01). The 18Lin^−^CD34^−^ cells formed erythroid (16.3%), megakaryocyte (29.3%) and erythro-megakaryocytic mixed colonies (54.2% Supplementary Figure S4B).

Finally, we analyzed the differentiation potentials of CD34^+/−^ HSPCs in the coculture system. The fold increase of 18Lin^−^CD34^+^ cells (207-fold) was significantly greater than that of 18Lin^−^CD34^−^ cells (29.9-fold; *P*<0.01)), after 7 days cocultuers (Supplementary Figure S5A). In addition, the number of CD34^+^ cells maintained/generated from 1 × 10^3^ 18Lin^−^CD34^+^ cells (4.6 × 10^4^) was significantly higher than that generated from 1 × 10^3^ 18Lin^−^CD34^−^ cells (0.6 x 10^4^) (*P*<0.01; Supplementary Figure S5B). The numbers of CD11b^+^ and CD14^+^ cells produced from one CD34^+^ cell generated from 1 × 10^3^ 18Lin^−^CD34^+^ cells were significantly higher than those produced from one CD34^+^ cell generated from 1 × 10^3^ 18Lin^−^CD34^−^ cells (*P*<0.01; Supplementary Figures S5D and E). Conversely, the number of CD41^+^ cells produced from one CD34^+^ cell generated from 1 × 10^3^ 18Lin^−^CD34^−^ cells was significantly higher than that produced from one CD34^+^ cell generated from 1 × 10^3^ 18Lin^−^CD34^+^ cells (*P*<0.01; Supplementary Figure S5F). These results are fairly consistent with the results of the CFC assay, in which 18Lin^−^CD34^−^ cells showed poor myeloid colony formation, and mainly formed erythro-megakaryocytic colonies.

It was recently reported that murine most primitive CD150^high^ or vWF^+^ HSCs showed megakaryocyte primed gene expression patterns, and in the CFC assay, they formed more megakaryocyte containing colonies compared with CD150^int^ or vWF^-^ HSCs.^3, 4, 5, 15^ Therefore, it is suggested that human CD34^−^ HSPCs are the human counterpart of the above-mentioned murine primitive HSCs.

In summary, the present data clearly demonstrated, for the first time, that human CB-derived CD34^−^ SRCs (HSCs) possess myeloid-biased LT-repopulating capacities. We also recently reported that CD34^−^ SRCs could produce most primitive CD34^+^ SRCs (Lin^−^CD34^+^CD38^−^CD90^+^CD45RA^−^ SRCs) in the cocultures with human bone marrow cell-derived mesenchymal stromal cells.^13^ These results suggest that myeloid-biased CD34^−^ HSCs may produce lymphoid-biased CD34^+^ HSCs. In other words, the expression of CD34 may segregate or separate lymphoid-biased HSCs from myeloid-biased HSCs. Collectively, it may be suggested that the human myeloid-biased LT-repopulating CD34^−^ HSCs reside in the apex of the human HSC hierarchy. However, the molecular mechanisms which control the lineage bias program of the primitive human HSCs have not yet been determined. Further investigation at the single-cell level is necessary to better elucidate the pathway of human HSC lineage differentiation.^15^ These studies also have important implications for clinical HSC transplantation in patients with malignant and nonmalignant hematological diseases.

## Figures and Tables

**Figure 1:**
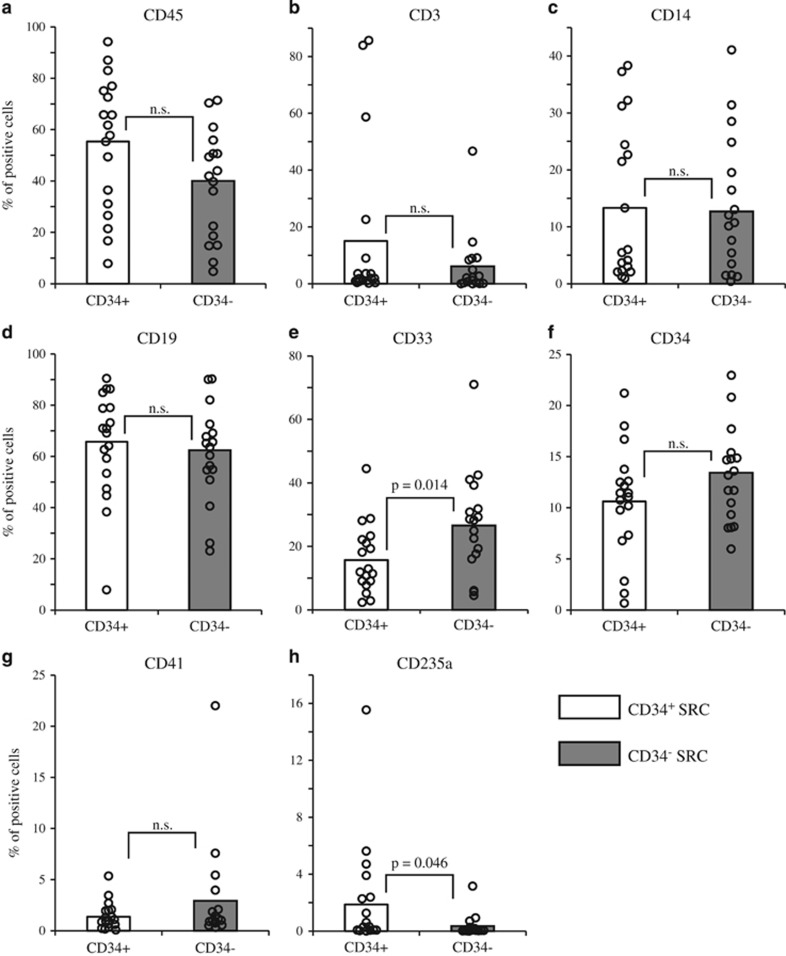
Comparision of the multi-lineage differentiation potentials between CD34^+/−^ SRCs at 18–24 weeks after transplantation. At 18–24 weeks after transplantation, the mice were killed and BM cells were collected from the left tibia. (**a**) The percentages of human CD45^+^ cells in the mouse BM with hemolysis. The expression of surface markers including human (**b**) CD3, (**c**) CD14, (**d**) CD19, (**e**) CD33, (**f**) CD34 and (**g**) CD41 on human CD45^+^ cells are indicated. (**h**) The percentages of CD235a^+^ cells on mouse CD45^−^ human CD45^+/−^ cells in the mouse BM without hemolysis. Each open circle represents the data of an individulal mouse. Open and gray bars show the mean percentages of each marker-positive cell.

**Figure 2:**
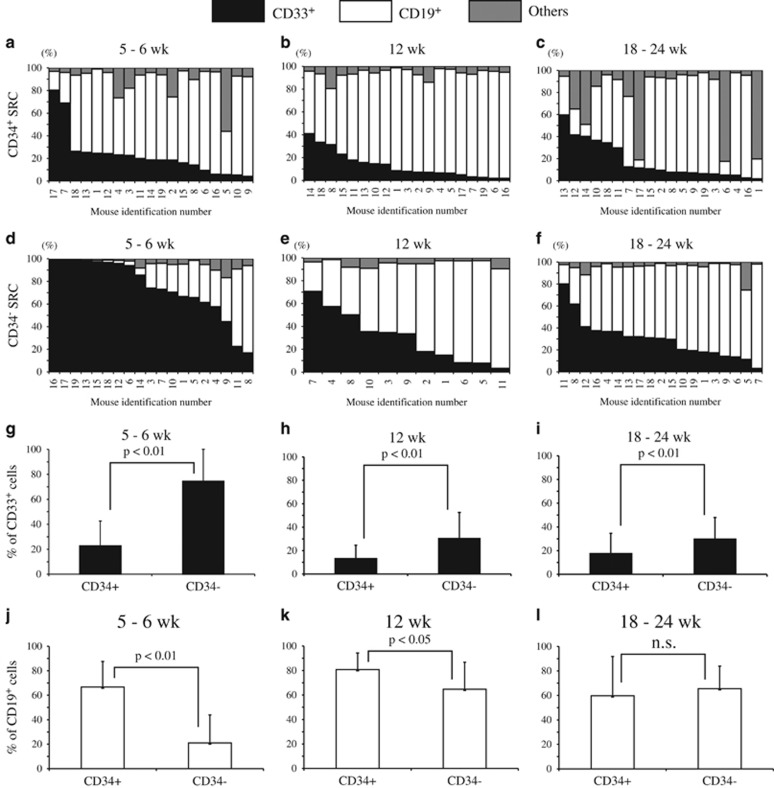
Serial analysis of the ratio of CD19^+^ and CD33^+^ cells produced from CD34^+/−^ SRCs in the mouse BM. The percentages of CD19^+^, CD33^+^ and other type of cells on the human CD45^+^ cells in the mice BM cells from the right tibia were serially analyzed from 5–24 weeks after transplantation by the BM aspiration method. The CD19^+^ or CD33^+^ cells on the human CD45^+^ cells were gated as indicated in Supplementary Figure S2. The human CD45^+^CD19^−^CD33^−^ cells were defined as ‘other type of cell'. Each individual mouse was identified by ear punching and the human hematopoietic cell repopulation was traced from 5–24 weeks after transplantation. (**a**–**f**) The percentages of CD33^+^ (filled bar), CD19^+^ (open bar) and other types of cells (gray bar) in the human CD45^+^ cells in the mouse BM from the right tibia. Each bar indicates the data of an individual mouse receiving (**a**–**c**) CD34^+^ and (**d**–**f**) CD34^−^ SRCs. The mean percentages of (**g**–**i**) CD33^+^ and (**j**–**l**) CD19^+^ cells on human CD45^+^ cells in the mouse BM at each time point are indicated. The data represent the means±s.d.
