# Supplementary material for: Human cord blood-derived primitive CD34-negative hematopoietic stem cells (HSCs) are myeloid-biased long-term repopulating HSCs
Source: Blood Cancer J. 2015 Mar 13;5(3):e290–. doi: 10.1038/bcj.2015.22 (PMC4382663; doi:10.1038/bcj.2015.22)
Supplement: Supplementary Information [file bcj201522x1.docx]

**Supplementary information for Matsuoka Y, et al.**

**Materials and Methods**

***Collection of the cord blood (CB) samples and processing of the lineage-negative (Lin^-^) cells***

All studies were approved by the Institutional Review Board of Kansai Medical University. Human CB samples were obtained from normal full-term deliveries with signed informed consent. The CB-derived Lin^-^ cells were prepared as previously reported.^1-3^ The CB mononuclear cells (MNC) were isolated using Ficoll-Paque PLUS (GE Healthcare Bio-sciences AB, Uppsala, Sweden). The CB MNCs were further enriched for lineage-negative cells using the EasySep Human Progenitor Cell Enrichment Kit (StemCell Technologies, Vancouver, BC, Canada) and manipulated using RoboSep (StemCell Technologies) according to the manufacturer’s instructions.

***Preparation of 18Lin^-^CD34^+/-^ cells***

Immunomagnetically-enriched CB-derived Lin^-^ cells were stained with fluorescein isothiocyanate (FITC)-conjugated 18 Lineage (Lin) mAbs against CD2, CD16, CD24, and CD235a (DAKO, Kyoto, Japan); CD3, CD7, CD10, CD11b, CD20, CD41, and CD66c (Beckman Coulter, Fullerton, CA); CD19 and CD56 (BD Biosciences, San Jose, CA); CD4, CD14, CD33 and CD127 (eBioscience, San Diego, CA); and CD45RA (Southern Biotech, Birmingham, AL); a Pacific Blue (PB)-conjugated anti-CD45 mAb (BioLegend, San Diego, CA); an allophycocyanin (APC)-conjugated anti-CD34 mAb (BD Biosciences) and 7-amino-actinomycin D (7-AAD) (Beckman Coulter, Fullerton, CA) as reported previously.^1-3^ The 18 Lin-negative (Lin^-^) CD34^+^ or 18Lin^-^CD34^-^ cells were sorted and collected using a BD FACSAria or FACSAriaIII cell sorter (Figure S1A).

***Hematopoietic colony-forming cell (CFC) assays***

The hematopoietic colony-forming cell (CFC) assays of freshly isolated 18Lin^-^CD34^+/-^ cells were performed according to our standard protocol for methylcellulose cultures as previously reported.^1-5^ One hundred 18Lin^-^CD34^+/-^ cells were seeded into a 35 mm culture dish in the presence of a cocktail of cytokines (SCF, IL-3, GM-CSF, G-CSF and Epo) plus 30% FCS or TPO, IL-3 and Epo plus 10% platelet-poor plasma. The number and types of CFCs were assessed on day 13 or day 10, respectively.

***Coculture of 18Lin^-^CD34^+/-^ cells with DP MSCs***

Cocultures of 18Lin^-^CD34^+/-^ cells with hematopoietic cell supportive mesenchymal stromal cells established from human bone marrow Lin^-^CD45^-^CD271^+^SSEA-4^+^ cells (DP MSCs) were performed as previously reported.^3^ DP MSCs were seeded on a 12-well culture plate (BD Biosciences) one week before starting the coculture. When the DP MSCs reached confluency, the cells were irradiated (12 Gy using a ^137^Cs-γ irradiator). Eighteen Lin^-^CD34^+^ (5.0 x 10^3^ cells per well) and CD34^-^ cells (1.5 x 10^4^ cells per well) were seeded onto DP MSC feeders and cocultured in StemPro-34 medium (Invitrogen) supplemented with a cocktail of cytokines, including 50 ng/mL stem cell factor (SCF) (BioScience Autogen, Wiltshire, UK), 50 ng/mL flt3 ligand (FL) (R&D Systems), 100 ng/mL thrombopoietin (TPO), 10 ng/mL interleukin (IL)-3 (R&D Systems), 100 U/mL IL-6 (kindly provided by Ajinomoto Co. Inc., Tokyo, Japan) and 10 ng/mL granulocyte colony-stimulating factor (G-CSF). G-CSF and TPO were kindly gifted by the Kyowa Hakko Kirin Company (Tokyo, Japan). After one week, all of the cells were collected by vigorous pipetting, and then, the cells were processed for the analysis.

After one week, the cocultured 18Lin^-^CD34^+/-^ cells with DP MSCs were collected and stained with PB-conjugated anti-CD45 mAb, APC-conjugated anti-CD34 mAb, FITC-conjugated anti-CD11b mAb, PE-conjugated anti-CD14 mAb, PE-conjugated anti-CD33 mAb, PE-conjugated anti-CD41 mAb and 7-AAD. The percentages of marker-positive cells in the viable CD45^+^ cells were analyzed using FACSCantoII (BD Biosciences). The absolute number of marker-positive cells was calculated from the total number of harvested cells and the percentages of lineage marker-positive cells.

***NOG mice***

Six-week-old female NOD/Shi-scid/IL-2Rγ_c_^null^ (NOG) ^6^ mice were purchased from the Central Institute of Experimental Animals (Kawasaki, Japan). All mice were handled under sterile conditions and were maintained in germ-free isolators located in the Central Laboratory Animal Facilities of Kansai Medical University. The animal experiments were approved by the Animal Care Committees of Kansai Medical University.

***SCID-repopulating cell (SRC) assays of 18Lin^-^CD34^+/-^ cells using an intra-BM injection (IBMI)***

The SRC assay and IBMI were carried out as reported previously.^1-5,7^ The mice were irradiated using a ^137^Cs-γ irradiator immediately before transplantation. Then, the mice were anesthetized with sodium pentobarbital. Five to 50 CD34^+^ SRCs or two to 35 CD34^-^ SRCs were transplanted into the left tibia of eight-week-old NOG mice (n = 19, each group). The number of SRCs contained in the 18Lin^-^CD34^+/-^ cells was calculated using our previous data.^1^ Each individual mouse was identified by ear punching and the human hematopoietic cell repopulation was traced from 5 to 24 weeks after transplantation. The repopulation kinetics of the human CD45^+^ hematopoietic cells in the BM from the right tibia were serially analyzed by the BM aspiration technique.^1-3,7^ BM aspiration was performed at 5 to 6, 12 and 18 to 24 weeks after transplantation. A portion of the mice receiving CD34^-^ SRCs were analyzed for the human CD45^+^ cell repopulation only 5 and 21 weeks after transplantation. The aspirated BM fluid was then diluted with PBS^-^/FCS plus 1% heparin. The collected BM cells were then stained with FITC-conjugated anti-CD19 mAb, PE-conjugated anti-CD33 mAb, APC-conjugated anti-CD34 mAb, PB-conjugated anti-human CD45 mAb, PE-Cy7-conjugated anti-mouse CD45.1 mAb (Beckman Coulter) and 7-aminoactinomycin D (AAD) (Beckman Coulter). The human hematopoietic cell repopulation in the murine BM was determined by the existence of human CD45^+^ cells in the 7-AAD^-^ living gate by six-color FCM. The mice that showed at least a 0.1% human CD45^+^ cell repopulation rate were analyzed through the entire assessment period. For the precise analysis of the differentiation potentials of CD34^+/-^ SRCs, the mice were sacrificed at18 to 24 weeks after transplantation and the BM cells were collected from the left tibiae by crushing in a mortar. The collected cells were suspended in PBS^-^/FCS and the remaining bone tissues were removed using a cell strainer (BD Falcon). Then, the cells were stained with FITC-conjugated anti-human CD3 (Beckman Coulter), CD19 (eBioscience), CD11b (Beckman Coulter) and CD235a (DAKO) mAbs; PE-conjugated anti-human CD33, CD14 and CD41 (Beckman Coulter); and an APC-conjugated anti-human CD34 mAb (BD Biosciences) for the detection of T and B-lymphoid, myeloid and human stem/progenitor hematopoietic cells.

***Statistical analysis***

In Figures 1, 2, S4 and S5 the differences in the means between each pair were examined by the two-tailed Student’s *t*-test. Statistical significance was considered to be present at P <0.05.

**Supplemental references**

1. Ishii M, Matsuoka Y, Sasaki Y, Nakatsuka R, Takahashi M, Nakamoto T *et al*. Development of a high-resolution purification method for precise functional characterization of primitive human cord blood-derived CD34-negative SCID-repopulating cells. *Exp Hematol* 2011; **39**:203-213.
2. Takahashi M, Matsuoka Y, Keisuke S, Nakatsuka R, Fujioka T, Kohno H *et al*. CD133 is a positive marker for a distinct class of primitive human cord blood derived CD34-negative hematopoietic stem cell. *Leukemia* 2014; **28**:1308-1315.
3. Matsuoka Y, Nakatsuka R, Sumide K, Kawamura H, Takahashi M, Fujioka T *et al*. Prospectively isolated human bone marrow cell-derived MSCs support primitive human CD34-negative hematopoietic stem cells. *Stem Cells* (in press).
4. Wang J, Kimura T, Asada R, Harada S, Yokota S, Kawamoto Y *et al*. SCID-repopulating cell activity of human cord blood-derived CD34^-^ cells assured by intra-bone marrow injection. *Blood* 2003; **101**:2924-2931.
5. Kimura T, Asada R, Wang J, Kimura T, Morioka M, Matsui K *et al*. Identification of long-term repopulating potential of human cord blood-derived CD34^-^flt3^-^ severe combined immunodeficiency-repopulating cells by intra-bone marrow injection. *Stem Cells* 2007; **25**:1348-1355.
6. Ito M, Hiramatsu H, Kobayashi K, Suzue K, Kawahata M, Hioki K, et al. NOD/SCID/γc^null^ mouse: an excellent recipient mouse model for engraftment of human cells. Blood. 2002;100:3175-3182.
7. Kimura T, Matsuoka Y, Murakami M, Kimura T, Takahashi M, Nakamoto T *et al*. *In vivo* dynamics of human cord blood-derived CD34^-^ SCID-repopulating cells using intra-bone marrow injection. *Leukemia* 2010; **24**:162-168.

**Legends for supplemental figures:**

**Figure S1. Schematic illustration of the serial analysis of the *in vivo* differentiation potentials of CD34^+/-^ SRCs in the mouse BM.**

(A) Isolation of human CB-derived 18Lin^-^CD34^+/-^ cells. The CD34^+^ fraction was determined according to the maximum APC fluorescence intensity (FI) to 5% the level of FI. The CD34^-^ level of FI was determined based on the Fluorescence Minus One controls as reported previously:^10-13^. (B) Schematic illustration of the SRC assay. Eighteen Lin^-^CD34^+/-^ cells were transplanted into the left tibia of irradiated (2.5 Gy) NOG mice by IBMI. At 5 to 6, 12 and 18 to 24 weeks after transplantation, the mice BM cells were harvested from the right tibia (non-injection site) by the BM aspiration technique. The human hematopoietic reconstitutions in the mice BM were analyzed by FACS. At 18 to 24 weeks after transplantation, the mice were sacrificed and the BM cells were collected from the left tibia (injection site), and the human hematopoietic cell reconstitutions were analyzed by FACS.

**Figure S2. The representative FACS profiles of multi-lineage differentiation of CD34^+/-^ SRCs at 18 to 24 weeks after transplantation.**

The representative FACS profiles of the human multi-lineage hematopoietic cell repopulation in two representative mice that received CD34^+/-^ SRCs are indicated. The data shows the human multi-lineage differentiation of hematopoietic cells in each individual mouse receiving (A) CD34^+^ SRCs and (B) CD34^-^ SRCs. Human CD45^+^ cells in the mouse BM with hemolysis were gated on R1. Then, the expression of CD34, CD19, CD33, CD3, CD41 and CD11b on human CD45^+^ cells were analyzed. For the detection of human erythrocytes in the mouse BM, the mouse CD45^-^ human CD45^+/-^ cells were gated on R2 without hemolysis. Then, the expression of CD235a on the mouse CD45^-^ human CD45^+/-^ cells was analyzed.

**Figure S3. Representative FACS profiles of human CD19^+^ and CD33^+^ cell reconstitution in the mouse BM receiving CD34^+/-^ SRCs at the early and late period after transplantations.**

The representative FACS profiles of each two mice receiving (A) CD34^+^ and (B) CD34^-^ SRCs are shown. CD19 and CD33 gates were set on 7AAD^-^ human CD45^+^ cells as indicated in Figure S2. The mouse identification numbers depicted in the left side of each plot correspond to those in Figure 2.

**Figure S4. CFC assay of 18Lin^-^CD34^+/-^ cells**

(A and B) The colony-forming capacities of sorted 18Lin^-^CD34^+/-^ cells are shown. One hundred sorted 18Lin^-^ CD34^+/-^ cells were cultured in methylcellulose at 37°C with 5% CO_2_, 5% O_2_ and 90% N_2_ in the presence of (A) SCF, TPO, IL-3, GM-CSF, G-CSF and EPO plus 30% FCS for 13 days or (B) TPO, IL-3 and EPO plus 10% platelet-poor plasma for 10 days. The types of colonies identified *in situ* were granulocytes (CFU-G), macrophages (CFU-M), granulocytes/macrophages (CFU-GM), erythroid burst (BFU-E), megakaryocytes (CFU-Meg), erythrocytes/megakaryocytes (CFU-EM) and an erythrocyte-containing mixed (CFU-Mix). Open, hatched, gray, closed and dotted bars represent the number of CFU-Mix, BFU-E, CFU-GM (including CFU-G, CFU-M, and CFU-GM), CFU-Meg and CFU-EM colonies, respectively. The numbers of all the types of hematopoietic colonies were determined as the mean of quadruplicate cultures. Error bars indicate the SD of the number of total colonies.

**Figure S5. *In vitro* lineage differentiation potentials of CD34^+/-^ HPCs in the coculture with DP MSCs.**

A total of 5 x 10^3^ of 18Lin^-^CD34^+^ (open bar) and 1.5 x 10^4^ of 18Lin^-^CD34^-^ cells (gray bar) were cocultured with DP MSCs in the presence of a cocktail of cytokines (TPO, SCF, FL, G-CSF, IL-3 and IL-6) for 7 days. (A) Fold increase and (B) absolute number of CD34^+^ cells maintained/generated from 1 x 10^3^ 18Lin^-^CD34^+/-^ cells. Absolute number of (C) CD33, (D) CD11b, (E) CD14 and (F) CD41-positive cells produced from one CD34^+^ cells generated from the 18Lin^-^CD34^+/-^ cells in the coculture. The expressions of each marker on the harvested cells were analyzed by FACS. The data shows the mean ± SD from two independent experiments (n = 6).
