# Supplementary figures and images for: Human cord blood-derived primitive CD34-negative hematopoietic stem cells (HSCs) are myeloid-biased long-term repopulating HSCs
Source: Blood Cancer J. 2015 Mar 13;5(3):e290–. doi: 10.1038/bcj.2015.22 (PMC4382663; doi:10.1038/bcj.2015.22)

(A)

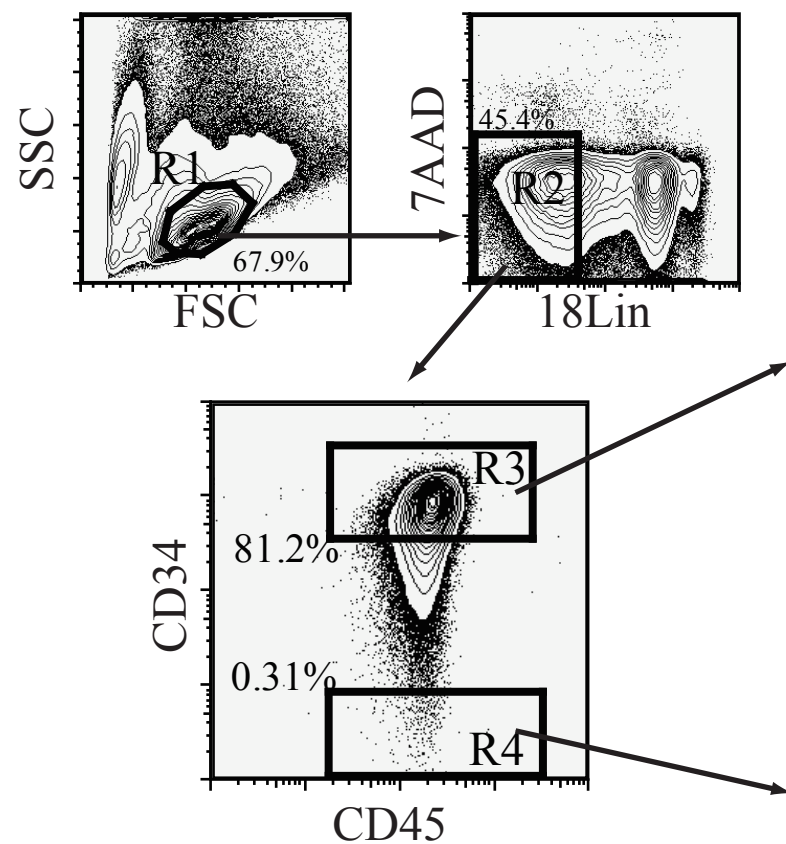

(B)

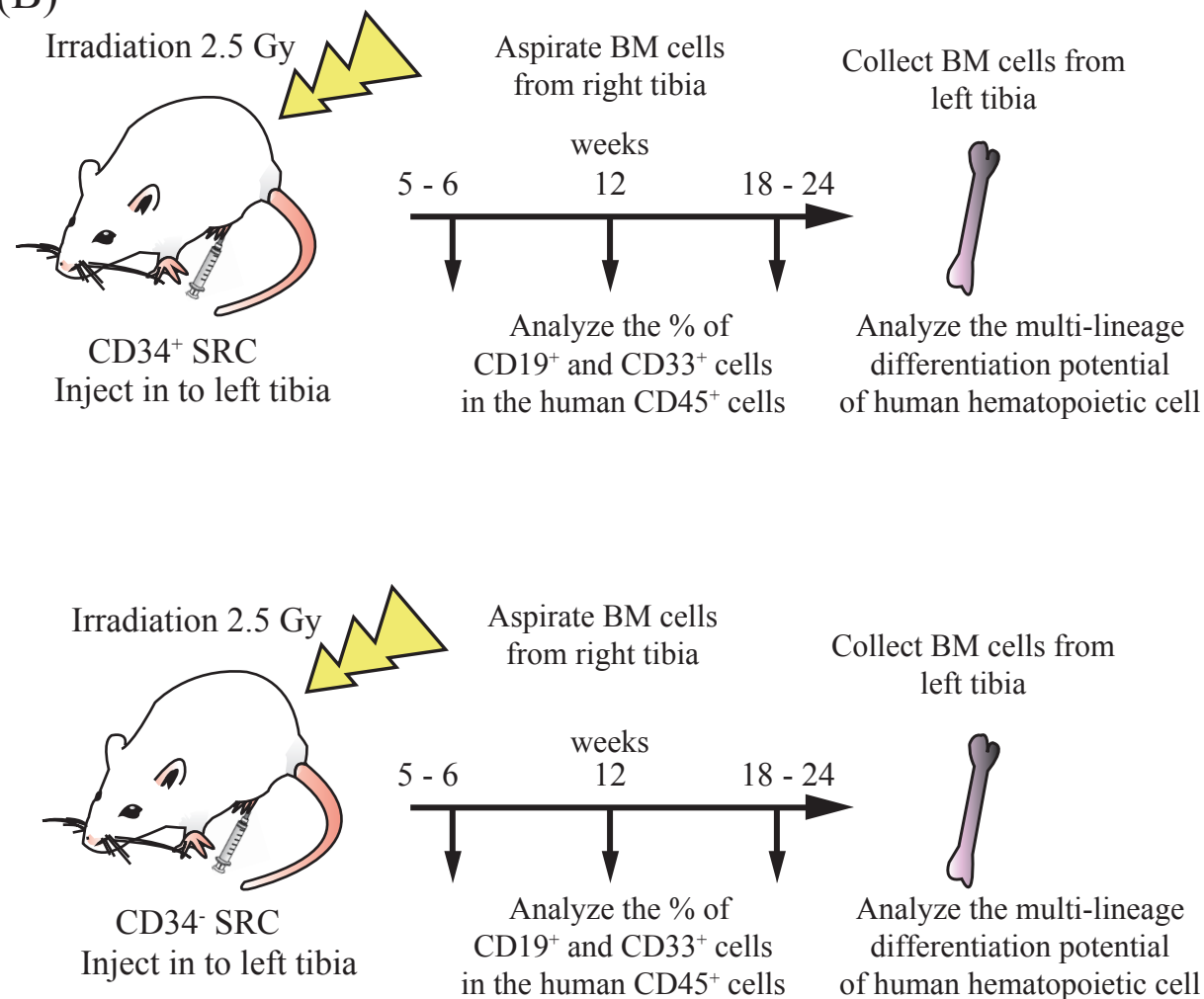

Supplement: Supplementary Figure S1 [file bcj201522x2.pdf]

(A) CD34<sup>+</sup> SRC

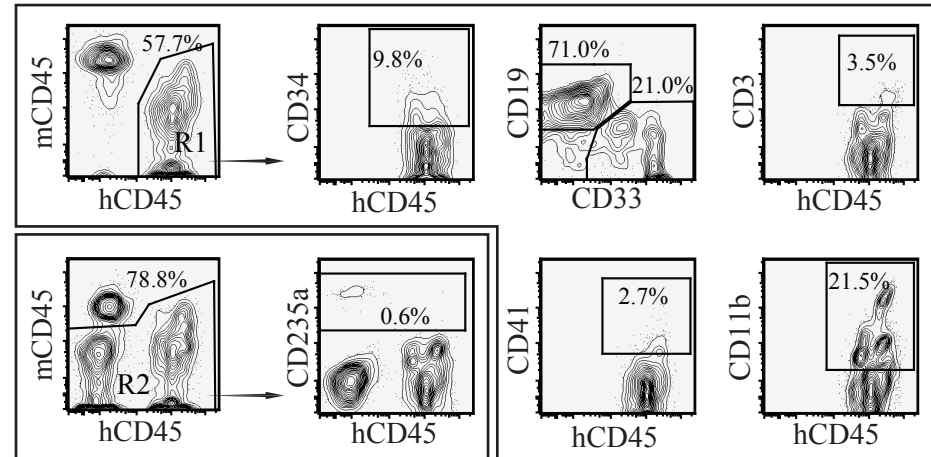

(B) CD34<sup>-</sup> SRC

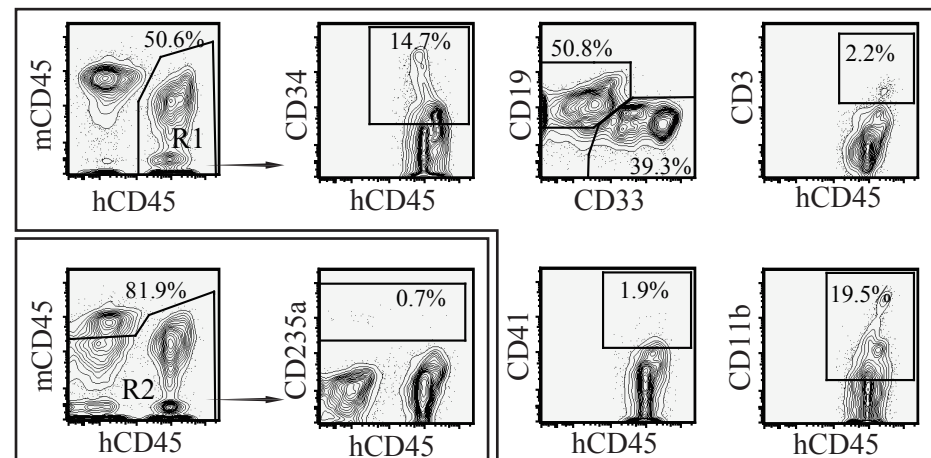

Supplement: Supplementary Figure S2 [file bcj201522x3.pdf]

(A) CD34<sup>+</sup> SRC

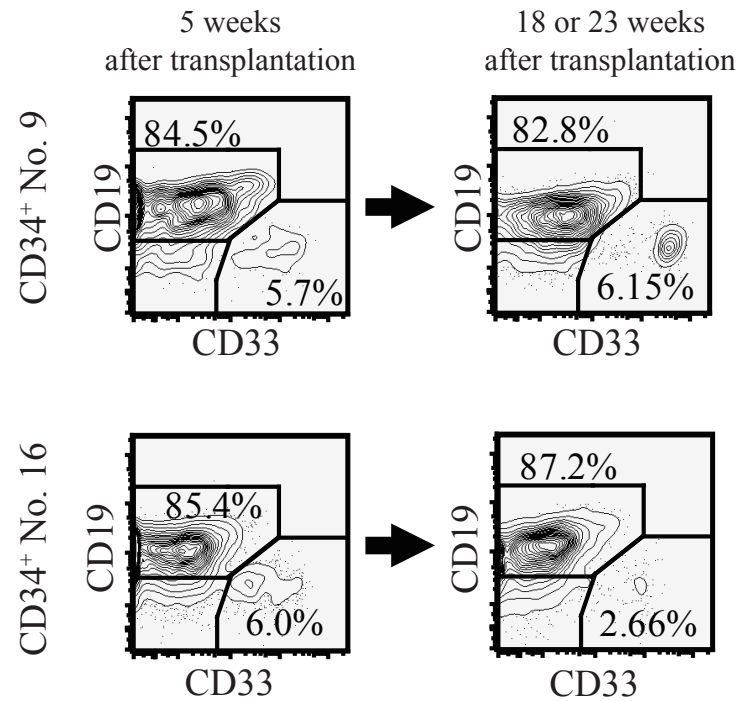

(B) CD34<sup>-</sup> SRC

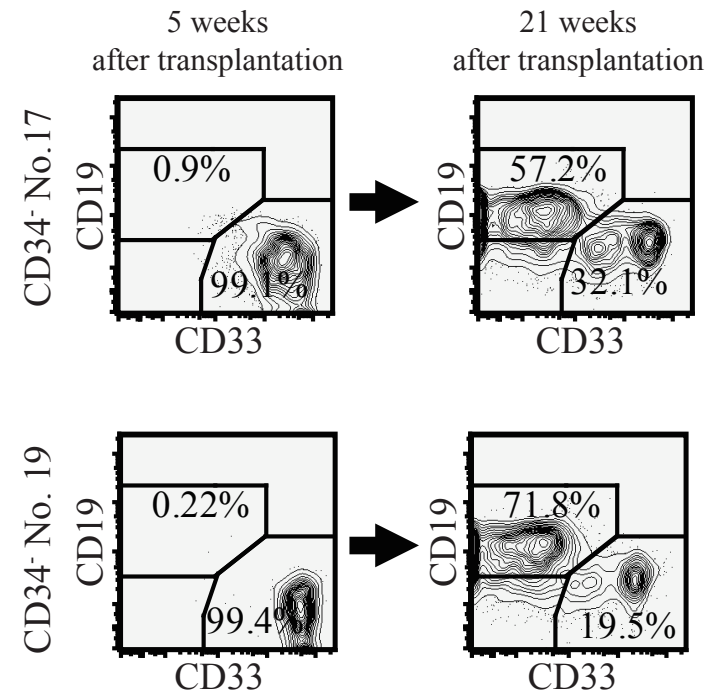

Supplement: Supplementary Figure S3 [file bcj201522x4.pdf]

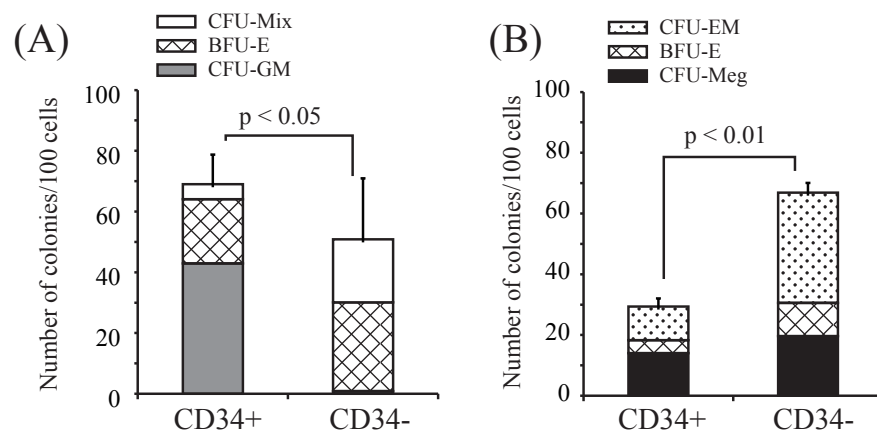

Supplement: Supplementary Figure S4 [file bcj201522x5.pdf]

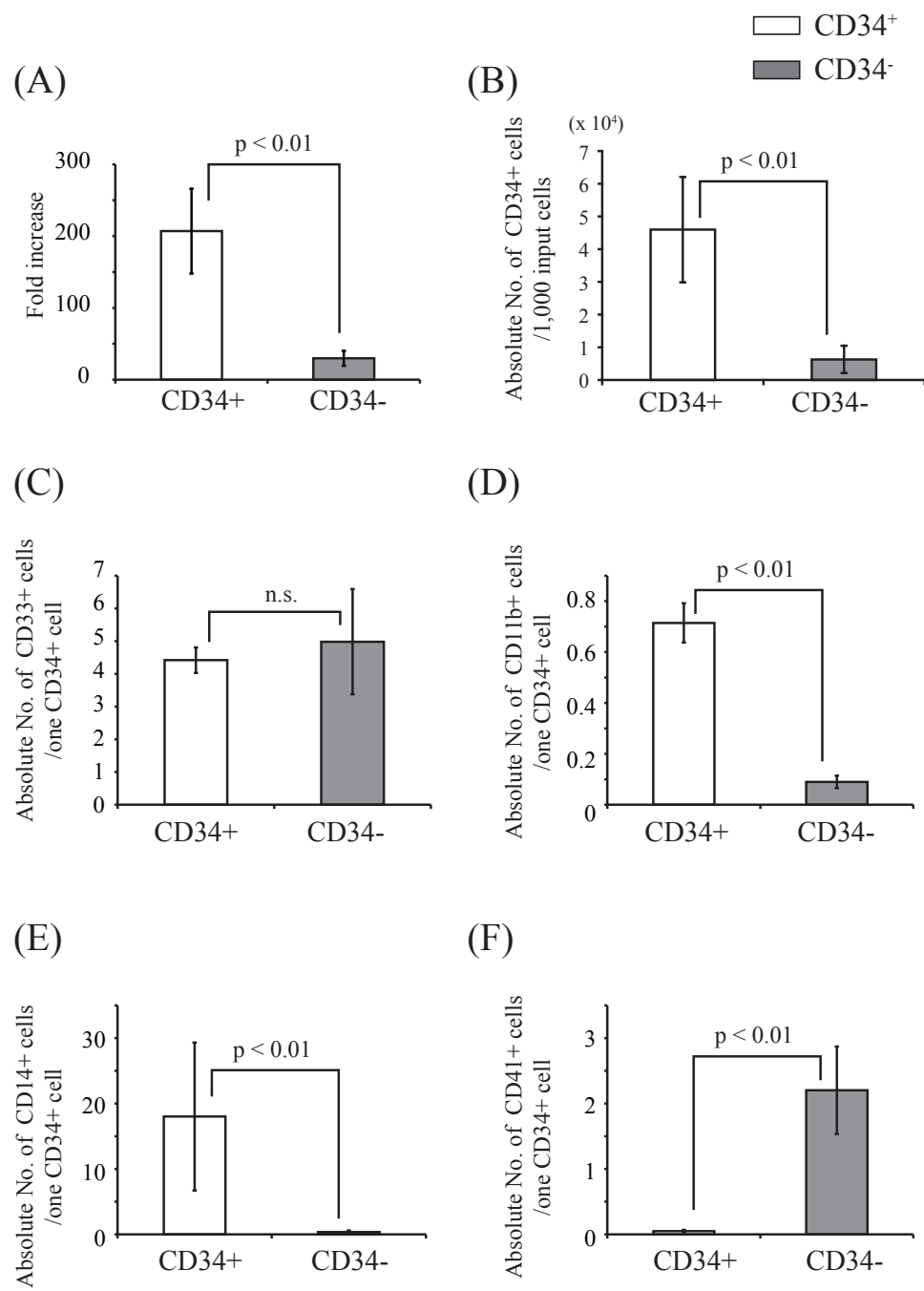

Supplement: Supplementary Figure S5 [file bcj201522x6.pdf]
